# Pathological Complete Response in a Rare Case of Rectal Small Cell Neuroendocrine Carcinoma Following Neoadjuvant Radiochemotherapy: A Case Report

**DOI:** 10.1002/ccr3.71464

**Published:** 2025-11-12

**Authors:** Abhisek Jha, Vivek Ghosh, Samit Gautam, Kuldeep Timilsina, Alina Rajbanshi, Pooja Yadav, Dhiraj Gupta, Asmita Rayamajhi, Narendra Pandit

**Affiliations:** ^1^ Department of Clinical Oncology Birat Medical College and Teaching Hospital Morang Nepal; ^2^ East West Medical College Dhaka Bangladesh; ^3^ Department of Pathology Birat Medical College and Teaching Hospital Morang Nepal; ^4^ Department of GI Surgery Birat Medical College and Teaching Hospital Morang Nepal

**Keywords:** abdominoperineal resection, neuroendocrine, radiochemotherapy, rectal bleed, small cell carcinoma

## Abstract

Rectal neuroendocrine neoplasms are rare, aggressive tumors comprising a small fraction of rectal malignancies but with increasing incidence due to improved endoscopic detection. Small cell neuroendocrine carcinoma, a high‐grade variant, poses significant diagnostic and therapeutic challenges due to its rapid progression and poor prognosis. We report a case of a 32‐year‐old male presenting with per‐rectal bleeding and pain for 6 months. Imaging revealed an anorectal mass with locoregional lymphadenopathy. Histopathology confirmed poorly differentiated small cell neuroendocrine carcinoma (Grade 3) via immunohistochemistry, with a high Ki‐67 index and neuroendocrine marker expression. The patient received neoadjuvant radiotherapy (59.4 Gray in 30 fractions over 6 weeks) with concurrent chemotherapy etoposide and cisplatin, followed by abdominoperineal resection. Postoperative histology showed a pathological complete response. This case underscores the importance of considering rectal neuroendocrine neoplasms in patients with persistent rectal symptoms and highlights the role of immunohistochemistry in diagnosis. Despite limited literature, our case supports the potential efficacy of neoadjuvant radiochemotherapy in tumor downstaging and improving surgical outcomes in poorly differentiated rectal neuroendocrine carcinoma. Early diagnosis, protocol‐based neoadjuvant radiochemotherapy, and appropriate surgical intervention can lead to favorable outcomes in high‐grade rectal neuroendocrine neoplasms. This case reinforces the need for awareness, guideline‐based management, and long‐term surveillance to optimize prognosis in these rare but aggressive tumors.

## Graphical abstract

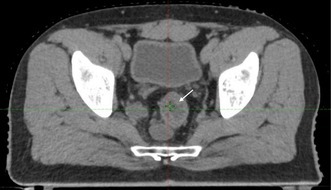

**Abbreviations**
^18^F‐FDG PET/CT: ^18^F‐fluorodeoxyglucose positron emission tomography/computed tomography
^68^Ga‐SSR‐PET/CT: gallium‐68 somatostatin receptor positron emission tomography/computed tomography
ENETS: European Neuroendocrine Tumor Society
NECs: neuroendocrine carcinomas
NENs: neuroendocrine neoplasms

**Summary**

- Adequately tailored neoadjuvant radiochemotherapy may offer significant tumor downstaging and the potential for complete pathological response in locally advanced rectal neuroendocrine neoplasms, supporting its consideration in multidisciplinary management.

## 1 Introduction

Rectal neuroendocrine neoplasms (NENs) are exceptionally rare rectal tumors with an incidence of 0.17% during screening colonoscopy even though they comprise 12%–27% of all NENs and 20% of gastrointestinal NENs. The increasing trend of rectal NENs in recent decades points toward the ever‐evolving endoscopic screening technique [1]. Rectal NENs usually present with vague symptoms such as perianal pain, blood per rectum, anemia, and perianal bulge [2]. According to the current European Neuroendocrine Tumor Society (ENETS) guidelines, well‐differentiated rectal NENs < 10 mm are recommended for endoscopic removal due to their low risk of local and distant invasion, whereas rectal NENs > 20 mm are indicated for surgical resection due to the higher risk of distant metastasis and involvement of the muscularis propria. Tumors between 10 and 20 mm still present an area of clinical uncertainty due to intermediate risk of metastasis as well as difficult endoscopic removal [3]. European Society for Medical Oncology practice guidelines also consider the role of medical therapy in the treatment of NENs, albeit only in Grade 3 NENs and recommend cisplatin or carboplatin with etoposide as standard first‐line chemotherapy in Grade 3 Neuroendocrine Carcinomas (NECs) [4]. We report an extremely rare case of a 32‐year‐old male with a 6‐month history of bleeding per rectum along with rectal pain which was revealed to be a poorly differentiated Small Cell Neuroendocrine Carcinoma Grade 3 on the basis of immunohistochemistry panel reports. It showed a remarkable clinical resolution following neoadjuvant radiotherapy with a cumulative dose of 59.4 Gray in 30 fractions over 6 weeks and 3 cycles of etoposide and cisplatin‐based concurrent chemotherapy. The patient underwent abdominoperineal resection (APR) 8 weeks post‐neoadjuvant therapy and postoperative histopathology revealed a pathological complete response. This case report highlights the importance of considering rectal neuroendocrine carcinomas as differentials for bleeding per rectum and also emphasizes the role of neoadjuvant radiotherapy in tumor shrinkage and the role of cisplatin and etoposide‐based chemotherapy in managing poorly differentiated rectal neuroendocrine carcinomas.

## 2 Case History/Examination

A 32‐year‐old male of South Asian origin presented to the oncology outpatient clinic with a history of per‐rectal bleeding for 6 months. Bleeding was insidious in onset and associated with pain per rectum. The patient also reported decreased appetite and 15% weight loss (from 65 to 55 kg) during the 6‐month period. The patient did not give a history of any other comorbid illnesses. The patient denied any history of addiction and family history was negative for malignancies. On clinical examination, the patient was pale, digital rectal examination revealed ulcero‐proliferative growth at 1.5 cm from the anal verge without frank bleed whereas the rest of the abdominal examination yielded normal findings. Reviews of the other systems were unremarkable.

## 3 Methods (Differential Diagnosis, Investigations, and Treatment)

**FIGURE 1:**
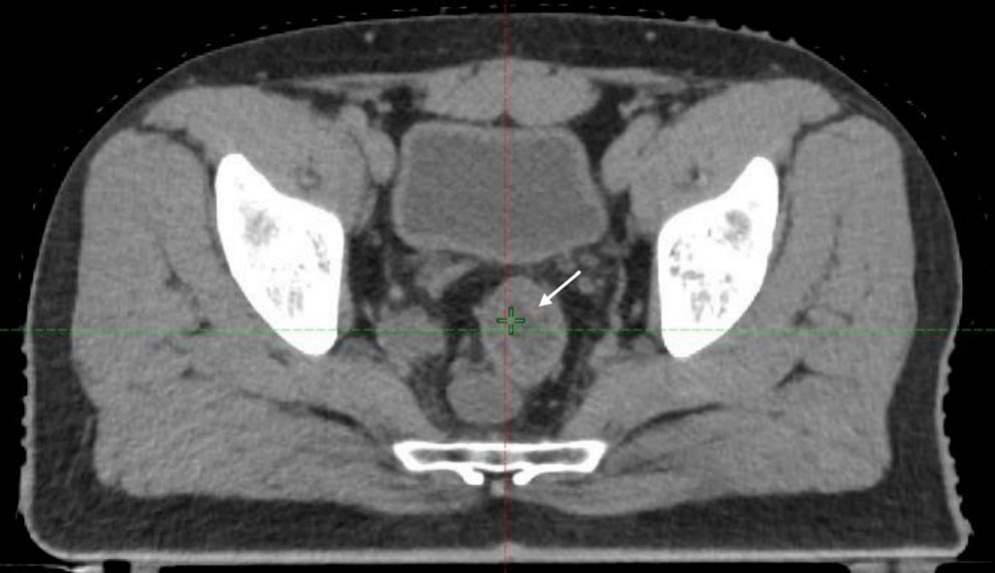
Contrast‐enhanced computed tomography of abdomen and pelvis showing heterogenously enhanced irregular circumferential thickening of anorectal wall with perirectal fat stranding (small white arrow) and multiple enlarged mesocolic and inguinal lymph nodes.

**FIGURE 2:**
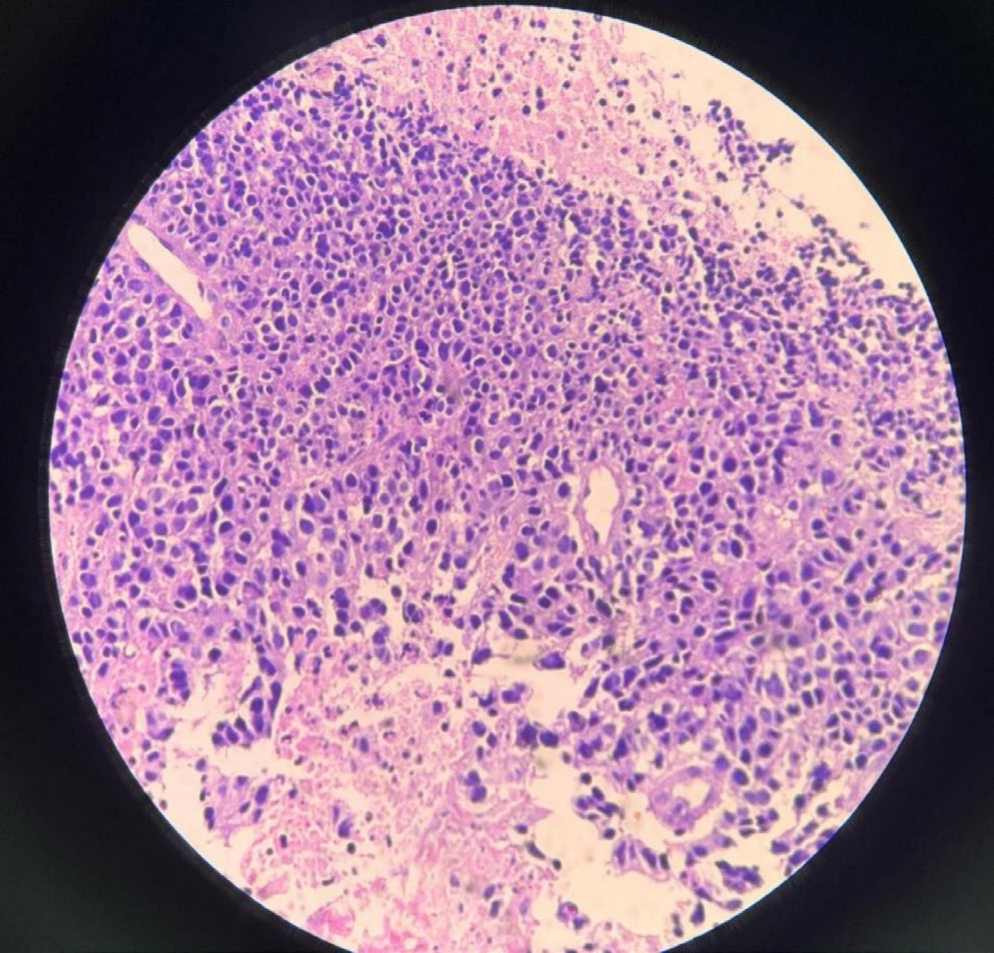
Histopathological image of rectal biopsy sample suggestive of poorly differentiated anorectal tumor.

Based on the history and thorough clinical examination, a suspicion of rectal tumor was made and the patient was planned for contrast‐enhanced computed tomography of the abdomen and pelvis. Preliminary hematological investigations were unremarkable except for low hemoglobin count—10.93 g/dL (normal range: 12–18 g/dL) and elevated carcinoembryogenic antigen (CEA)—8.6 ng/mL (Normal range: < 2.5 ng/mL). The imaging showcased a heterogeneously enhanced irregular circumferential thickening of the anorectal wall measuring 7 × 13 mm in thickness, 5.7 cm in length, and about 1.7 cm from the anal verge leading to luminal narrowing with perirectal fat stranding abutting the prostate. The scan also revealed multiple enlarged mesocolic lymph nodes ranging from 12 to 21 mm in short axis diameter, with a solitary necrotic right inguinal node measuring 20 mm (Figure 1). To further delineate the nature of the lesion, colonoscopy‐guided biopsy was performed. Colonoscopy demonstrated a distal friable mass in the rectum with a normal anal canal and the rest of the colon whereas the biopsy was suggestive of poorly differentiated malignant neoplasm of the rectum (Figure 2). Immunohistochemistry was further advised for exact histogenesis of the tumor and further treatment of the condition. The immunohistology reports outlined features suggestive of small cell neuroendocrine carcinoma (Table 1). In accordance with the standard treatment protocols, the patient was planned for neoadjuvant radiochemotherapy. The patient received a cumulative radiation dose of 59.4 Gray (Gy) in 30 fractions over 6 weeks and 3 cycles of etoposide and cisplatin‐based concurrent chemotherapy. He was treated with intensity modulated radiotherapy technique in two phases including 45 Gy in 25 fractions to the entire pelvis and inguinal regions followed by a boost to the gross tumor and involved nodes. He had grade two radiation dermatitis grade two diarrhea during the course of neoadjuvant therapy. The patient then underwent abdominoperineal resection for tumor removal along with end sigmoid colostomy for bowel functioning after 8 weeks of completion of neoadjuvant therapy.

**TABLE 1:** Immunohistochemistry panel reports suggestive of small cell neuroendocrine carcinoma.

IHC marker	Immunoreactivity	Score/Pattern
CK4	Positive in most tumor cells	4+
CD45	Negative in tumor cells	Score 0
S‐100	Negative in tumor cells	Score 0
Synaptophysin	Positive in most tumor cells	4+
Ki‐67	Positive in 99%–100% of tumor cells	High proliferation index
CGA (Chromogranin A)	Positive in most tumor cells	4+
INSM‐1	Positive in most tumor cells	4+
p53	Immunoreactivity in null mutant pattern	Abnormal

The patient is planned for a further three cycles of adjuvant Etoposide and Cisplatin and follow‐up thereafter.

## 4 Results and Conclusions (Outcome and Follow‐Up)

**FIGURE 3:**
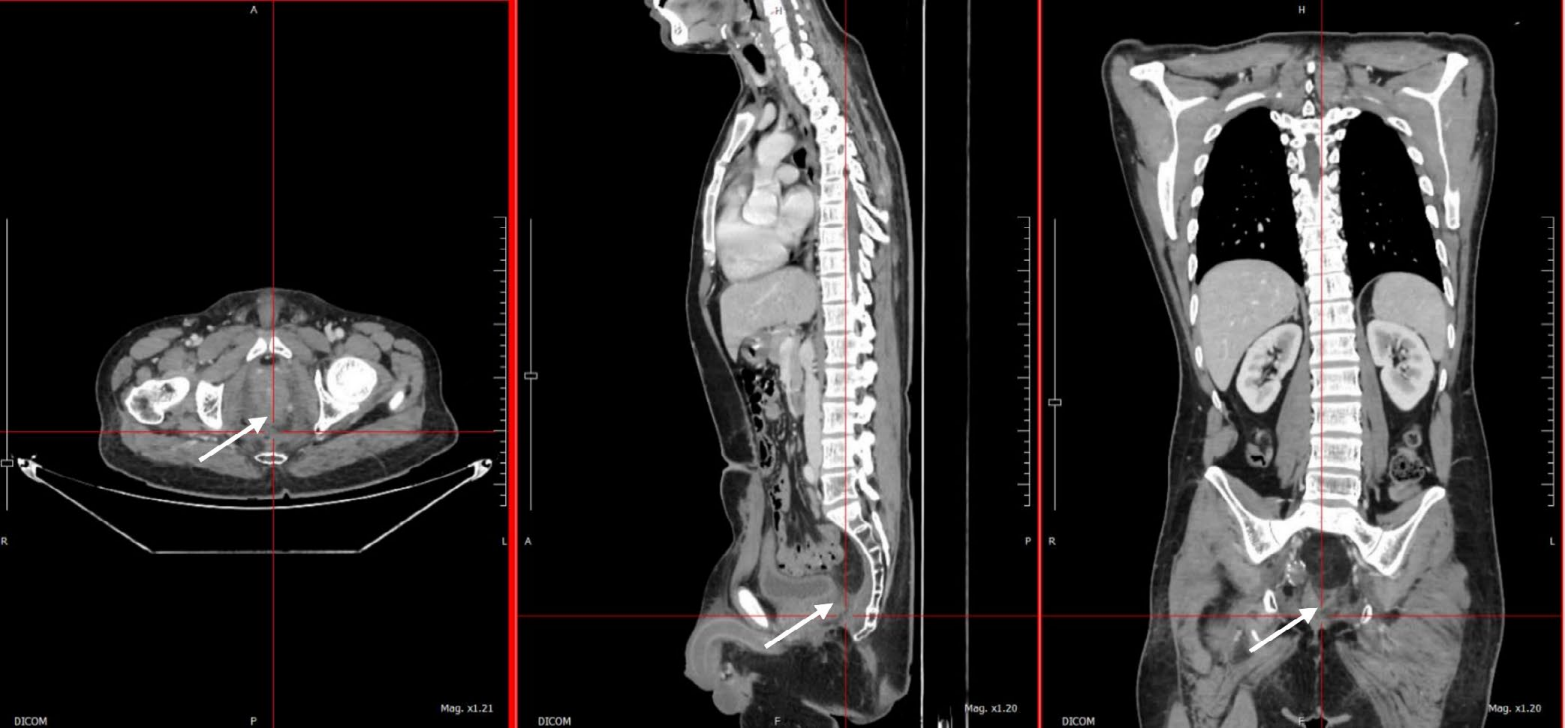
Contrast‐enhanced computed tomography of abdomen and pelvis showing no abnormal enhancement at the operation site with mildly enhancing thickening in the presacral region—likely postoperative changes without any evidence of disease recurrence and metastasis in a known case of rectal carcinoma (post‐radiochemotherapy and postoperative status).

Given the nature of the disease, the patient is on regular follow‐up and showed remarkable improvement in symptoms with only mild signs of radiation toxicity such as transient fatigue, dermatitis, diarrhea, and drop in cell counts. Also, the hematological toxicities were within acceptable limits. There were no unplanned treatment interruptions. The surgery was also well tolerated with improvement in weight gain along with a well‐functioning stoma. The postoperative histopathology reports showed a pathological complete response whereas the postoperative computed tomography imaging at 6 months follow‐up demonstrated no abnormal enhancement at the operation site with mildly enhancing thickening in the presacral region—likely postoperative changes without evidence of any recurrence and metastasis (Figure 3). The patient will undergo regular imaging studies as a part of a long‐term surveillance plan.

In conclusion, the response of the rectal neuroendocrine carcinoma was excellent to the neoadjuvant radiochemotherapy and the surgery and his prognosis is optimistic. The timely recognition of the condition with appropriate and protocol‐based treatment was of paramount importance in managing the condition with a favorable outcome. This case emphasizes the importance of considering rectal neuroendocrine carcinomas as a differential for chronic per‐rectal bleeding and advocates for guideline‐based treatment in the form of neoadjuvant therapy and appropriate surgery for better outcomes and fairer prognosis.

## 5 Discussion

Neuroendocrine carcinomas are rare malignancies arising from the neuroendocrine cells. Though these cells are found throughout the body, the most common site for these tumors is the gastrointestinal tract. Rectal NETs account for up to 27% of all NETs and 20% of gastrointestinal tract NETs while the rectal NETs account for only 1% of all rectal neoplasms. There has been an increasing trend in the incidence of colorectal NETs owing to advancing endoscopic technology and increased detection rates [5]. This increased disease burden necessitates further review of these understudied and rare tumors and increased awareness among physicians regarding the presentation, diagnosis, and management of neuroendocrine carcinomas.

Rodrigues et al. [6] report that patients with rectal neuroendocrine carcinomas are usually asymptomatic, whereas symptomatic patients present with vague rectal complaints such as rectal pain, bleeding, and tenesmus. In line with the above literature, in this case as well, the patient presented with chronic rectal bleeding and pain during defecation, highlighting the importance of considering rectal neuroendocrine carcinomas as a possible cause of ambiguous rectal complaints.

The diagnosis of rectal neuroendocrine carcinoma heavily depends on histological features as endoscopic characteristics can be misleading. On colonoscopy, a rectal neuroendocrine carcinoma usually appears as small, round polypoid lesions characterized by a smooth, normal‐appearing, or yellow‐discolored mucosa with a round shape pit pattern. However, atypical endoscopic appearances such as semi‐pedunculated appearance, hyperemia, central depression, erosion, and ulceration are usually seen in tumors of diameter > 5 mm [7]. In this context, the friable nature of the mass on colonoscopy deviates from the usual presentation of rectal neuroendocrine carcinoma, complicating the diagnostic process. European Society for Medical Oncology practice guidelines mandate histological diagnosis in all the patients and suggest the reporting of morphology, grading, and immunohistochemical staining of chromogranin A (cgA) and synaptophysin for appropriate pathological diagnosis of the tumor. Ki‐67 holds prognostic significance whereas p53 has a role in distinguishing G3 rectal neuroendocrine carcinomas from poorly differentiated NECs. Computed tomography constitutes the basic radiological method to image neuroendocrine tumors due to its wide availability, standardized reproducible technique and generally high diagnostic yield with sensitivity ranging from 61% to 93% and specificity from 71% to 100% [4]. Consistent with the above practice guideline, for the evaluation of the extent, spread and lymphovascular involvement of the tumor, we did Contrast‐enhanced Computed Tomography of the Abdomen and Pelvis which demonstrated irregular circumferential thickening of the rectal wall with involvement of mesocolic nodes. Immunohistochemical analysis was instrumental in determining the nature of the tumor as it demonstrated strong neuroendocrine markers expression and a high Ki‐67 index (99%–100%), consistent with high‐grade neuroendocrine carcinoma. Negative CD45 and S‐100, with abnormal p53, indicated nonlymphoid origin and aggressive behavior.

Once a lesion is identified as a neuroendocrine tumor, the choice of resection technique depends on tumor size, grade, and lymph node involvement. European Neuroendocrine Tumor Society (ENETS) guidelines recommend endoscopic resection for rectal neuroendocrine carcinomas ≤ 10 mm due to low locoregional and distant metastasis risk, oncological resection for tumors > 20 mm due to increased risk of distant metastasis, and individualized management for tumors between 10 and 20 mm based on preoperative evaluation and multidisciplinary input [3]. Lesions ≥ 20 mm or with lymph node metastases require oncological surgery—either anterior resection or abdominoperineal resection with total mesorectal excision—due to higher metastatic risk and require proper counseling as these surgeries carry the potential for significant comorbidities. Furthermore, a watch‐and‐wait strategy should only be adopted where resection is contraindicated or due to patient preference after appropriate counseling [8]. In line with the ENETS guideline, and as there were no contraindications to surgery, our patient underwent abdominoperineal resection with total mesorectal excision with end sigmoid colostomy after proper counseling at 8 weeks of completion of neoadjuvant therapy, as the tumor was 1.7 cm from the anal verge, 5.7 cm in length, 7 × 13 mm in thickness with mesocolic nodal involvement.

European Society for Medical Oncology practice guidelines explain no role of adjuvant therapy in benign NETs, that is, Grade 1/Grade 2. However, in aggressive NENs (NEC Grade 3), the guideline considers the role of platinum‐based chemotherapy [4]. Though the literature demonstrating the benefit of neoadjuvant radiochemotherapy in rectal NETs is sparse, several case reports mention the efficacy of neoadjuvant therapy in near complete response and tumor size reduction [9]. A case report by Voong and colleagues showcases that the patients who had preoperative radiochemotherapy only had a microscopic focus of residual carcinoma at surgery thereby highlighting the efficacy of radiochemotherapy in both tumor shrinkage and metastasis control [10]. In a similar vein, our patient also reported remarkable reduction in tumor size post platinum‐based chemotherapy in the form of Cisplatin and Etoposide and radiotherapy.

The survivability and prognosis of rectal neuroendocrine carcinomas are grim due to their aggressive nature. Metastatic rectal neuroendocrine carcinomas tend to have a poor prognosis despite appropriate platinum‐based chemotherapy [11]. However, in this scenario, the tumor spread was confined to the regional lymph nodes without any signs of distant metastasis. Post‐neoadjuvant radiochemotherapy, there was a significant reduction in the tumor volume impacting both survivability and prognosis.

The surveillance and follow‐up criteria for rectal neuroendocrine carcinomas is still in its nascent stage. Follow‐up after rectal neuroendocrine carcinomas resection depends on tumor grade, size, margin status, and lymphovascular invasion. No surveillance is needed for R0 resection of Grade 1 tumors ≤ 10 mm without lymphovascular invasion. For higher grade or lymphovascular invasion positive tumors ≤ 10 mm, and all tumors > 10 mm, 6‐monthly Magnetic Resonance Imaging, annual sigmoidoscopy, and ^68^Ga‐SSR‐PET/CT at baseline and 12 months are advised. R1 resections require yearly endoscopy and imaging. Grade 3 tumors warrant intensive imaging and endoscopic follow‐up, with consideration of ^18^F‐FDG PET/CT [3]. However, these follow‐up criteria are very stringent, economically demanding and very far from reality in a low socioeconomic region such as ours. In our case, the short follow‐up period has been a major limitation in the study. However, the patient has not shown the recurrence of the disease and the features of metastasis on 6‐month follow‐up CT imaging and is under regular follow‐up.

## 6 Conclusion

Rectal neuroendocrine tumors comprise only a smaller fraction of rectal carcinomas. Though rare, due to their increasing incidence and vague rectal presentations, they should be included in the differential diagnosis of chronic rectal bleed. This report aims to raise awareness among physicians and surgeons regarding the varied clinical presentation of neuroendocrine carcinomas and advocates for appropriate imaging and histochemical analysis for the timely diagnosis and proper treatment planning of neuroendocrine tumors. This case report emphasizes the efficacy of neoadjuvant radiochemotherapy in reducing the tumor burden and improving overall survivability and offering a better prognosis in neuroendocrine carcinomas. This text also underscores the importance of regular follow‐up and a long‐term surveillance plan to assess recurrence, locoregional spread and metastasis of the disease and timely plan the intervention in order to improve survivability and prognosis.

## Author Contributions

**Abhisek Jha:** conceptualization, writing – original draft. **Vivek Ghosh:** conceptualization, resources, supervision, writing – original draft, writing – review and editing. **Samit Gautam:** data curation, writing – review and editing. **Kuldeep Timilsina:** writing – review and editing. **Alina Rajbanshi:** writing – review and editing. **Pooja Yadav:** investigation. **Dhiraj Gupta:** writing – review and editing. **Asmita Rayamajhi:** supervision. **Narendra Pandit:** supervision.

## Consent

Written informed consent was obtained from the patient to publish this report in accordance with the journal's patient consent policy.

## Conflicts of Interest

The authors declare no conflicts of interest.

## Data Availability

Data will be provided by the corresponding author upon request. Images will be provided via a separate file.
